# West-Life: A Virtual Research Environment for structural biology

**DOI:** 10.1016/j.yjsbx.2019.100006

**Published:** 2019-02-26

**Authors:** Chris Morris, Paolo Andreetto, Lucia Banci, Alexandre M.J.J. Bonvin, Grzegorz Chojnowski, Laura del Cano, José Marıa Carazo, Pablo Conesa, Susan Daenke, George Damaskos, Andrea Giachetti, Natalie E.C. Haley, Maarten L. Hekkelman, Philipp Heuser, Robbie P. Joosten, Daniel Kouřil, Aleš Křenek, Tomáš Kulhánek, Victor S. Lamzin, Nurul Nadzirin, Anastassis Perrakis, Antonio Rosato, Fiona Sanderson, Joan Segura, Joerg Schaarschmidt, Egor Sobolev, Sergio Traldi, Mikael E. Trellet, Sameer Velankar, Marco Verlato, Martyn Winn

**Affiliations:** a STFC, Daresbury Laboratory, Warrington, UK; b INFN Padova Division, Italy; c Magnetic Resonance Center, University of Florence, Italy; d Utrecht University, The Netherlands; e National Center for Biotechnology, Spain; f Instruct-ERIC, Oxford, UK; g Division of Biochemistry, Netherlands Cancer Institute, Amsterdam, The Netherlands; h Masaryk University, Czech Republic; i European Molecular Biology Laboratory, c/o DESY, Notkestr. 85, 22607 Hamburg, Germany; j European Molecular Biology Laboratory (EMBL), European Bioinformatics Institute (EMBL-EBI), Cambridge, UK

**Keywords:** Structural biology, Virtual Research Environment, Cloud computing, Grid computing, Data management

## Abstract

The West-Life project (https://about.west-life.eu/) is a Horizon 2020 project funded by the European Commission to provide data processing and data management services for the international community of structural biologists, and in particular to support integrative experimental approaches within the field of structural biology. It has developed enhancements to existing web services for structure solution and analysis, created new pipelines to link these services into more complex higher-level workflows, and added new data management facilities. Through this work it has striven to make the benefits of European e-Infrastructures more accessible to life-science researchers in general and structural biologists in particular.

## Highlights

• Data processing and data management services for structural biology.
• Enhancements to existing web services for structure solution and analysis.
• New pipelines to link these services into more complex higher-level workflows.
• New data management facilities.
• Making the benefits of European e-Infrastructures more accessible to structural biologists.

## Graphical abstract

**Figure:**
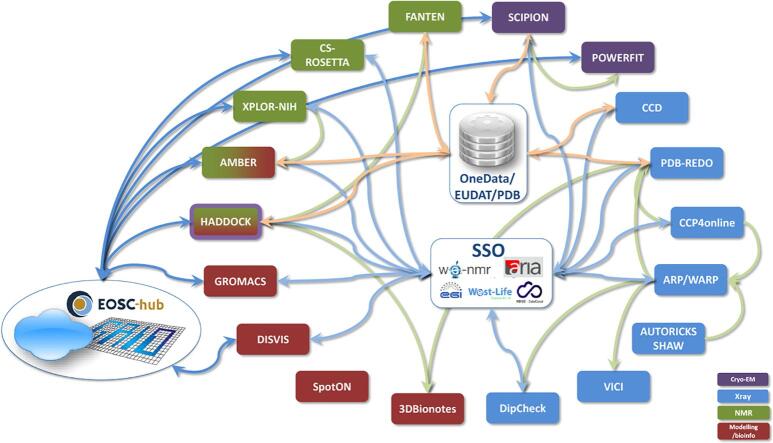

## 1 Introduction

Recent advances in structure determination techniques have made it possible to investigate large multi-component macromolecular machines rather than single gene products. Traditionally structure determination is carried out using one or a few experimental techniques, for example Macromolecular X-ray crystallography (MX), Nuclear Magnetic Resonance (NMR), or Electron cryo-Microscopy (cryo-EM), but a full characterisation of large macromolecular machines often requires a combination of several structural and computational methods.

**Fig. 1:**
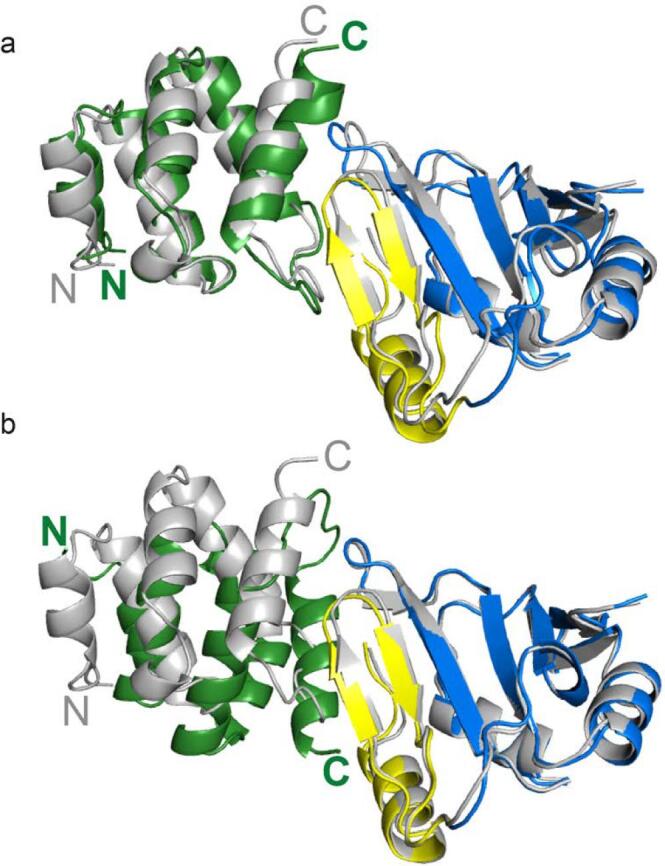
Cartoon representation of the two main families from HADDOCK docking of the NURR and eRRM1 domains of the RNA-binding protein Syncrip, superimposed on the crystal structure (Hobor et al., 2018).

For example a recent study of the RNA-binding protein Syncrip by Hobor et al. used several NMR methods, X-ray crystallography, Biolayer interferometry, and UV-crosslinking RNA Immuno-Precipitation, along with computational methods (Hobor et al., 2018). Fig. 1 shows results obtained using the HADDOCK service provided by West-Life.

**Fig. 2:**
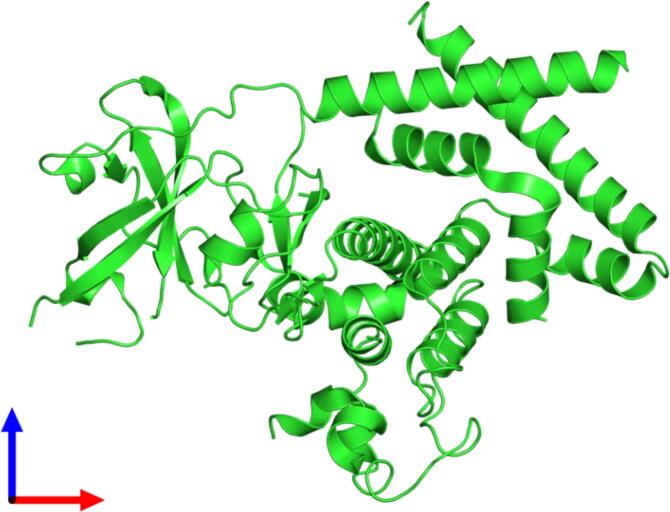
The crystal structure of pseudokinase PEAK1 (Ha and Boggon, 2017), obtained through a combination of CCP4 online pipelines and the ARP/wARP web-service.

An example of a synergy between the ARP/wARP webservice and CCP4online tools, both supported within the West-Life project, is given by the crystal structure determination of PEAK1 pseudokinase (Ha and Boggon, 2017), shown in Fig. 2. Following a number of unsuccessful molecular replacement attempts, an initial phasing solution was obtained using the CCP4 molecular replacement pipeline Balbes (Long et al., 2008). Due to the relatively weak molecular replacement solution, a highly-complete model could only be obtained after multiple rounds of model building with the ARP/wARP web-service, varying parameters for the free-atoms update. The best model from each building round was re-input to ARP/wARP allowing 365 residues (85% of the sequence) to be built. The model was subsequently completed and refined using standard crystallographic protocols.

The information technology infrastructure that supports these researchers must keep pace with new demands. It must provide implementations of algorithms that can integrate data from different methods, and help track the processing steps and data in these increasingly complex projects (Morris, 2013). This e-infrastructure should ideally be provided as web services with user-friendly interfaces. While, for example, an experienced crystallographer may easily install and use the CCP4 suite (Winn et al., 2011), a researcher undertaking crystallography for the first time may find installation of the full suite a deterrent to use, and may also be uncertain how to navigate through the suite. The CCP4online web services minimise these issues and provide an easy route to much of the functionality, though the downloadable suite will always be more fully featured.

Instruct-ERIC, the European infrastructure for structural biology, encourages such use of combined techniques by facilitating and funding visits to experimental facilities, and providing training to researchers learning new techniques. West-Life, a European Union Horizon 2020 e-Infrastructure Virtual Research Environment, is designed to facilitate the use of the associated computational methods. It does this through a set of user-facing services aimed at the international community of structural biologists, and also via infrastructure tools aimed at the community of service developers. This paper summarizes these developments.

*Related Work* Since around 2010, the WeNMR consortium (http://www.wenmr.eu/) has been providing a range of web services focused around NMR (Wassenaar et al., 2012). Most services make use of the European Grid Infrastructure (EGI,http://www.egi.eu/) federated high-throughput computing (HTC) resources. This enables the computational load to be distributed across over 40 European, Asian and US sites, which support the enmr.eu Virtual Organisation and provide access to over 100,000 CPU cores. Several partners in West-Life are also partners in WeNMR, which forms an identifiable subset of the broader West-Life community. West-Life has continued to support and improve the WeNMR services, and has promoted links to MX and cryo-EM services through collaboration with other major European software developers.

## 2 Results and discussion

The West-Life Virtual Research Environment (VRE) provides a complete environment for analysing structural biology data. As such, it consists of multiple components handling data processing, data management, compute resources, infrastructure for authentication and authorisation, quality assurance and user help. In the following sections, we give a brief overview of these components. *Portal* describes the entry point for West-Life users, giving access to all services and support (the current service provision is shown in Table 1). *Enhanced Services*, *Quality Assurance* and *Pipelines* describe extensions to service functionality and interoperability developed by the West-Life project. *Data Management* and *Metadata* describe tools for handling and annotating structural biology data. Finally, *Single Sign On*, *EGI*, and *Virtualized Portals* describe technological aspects of the VRE.

**Table 1:** Current portfolio of West-Life Services. The principal scientific domain is listed, though services increasingly span multiple domains. Direct links between services are listed explicitly. The final column summarises the enhancements implemented by West-Life, where EM: extension to cryoEM datasets, SSO: Single Sign On, GPU: port to graphics cards.

Service	Domain	Links	W-L Contribution
Haddock	Integrative Modelling/ NMR/ EM	FANTEN, 3DBioNotes	EM, SSO, links
DisVis	Integrative Modelling		GPU, SSO
Powerfit	Integrative Modelling/ EM	Scipion	GPU, server version, links
SpotOn	Integrative Modelling		new service
PRODIGY(-LIG)	Integrative Modelling		version for ligands
DipCheck	Integrative Modelling	ARP/wARP	new service, SSO, links
Scipion Web Tools	Electron Microscopy	PowerFit	new services, Virtual Folder, links, cloud
CS-ROSETTA3	NMR		SSO
AMPS-NMR	NMR	Xplor-NIH	GPU, SSO, links
FANTEN	NMR	HADDOCK	Virtual Folder, links
Xplor-NIH	Integrative Modelling	AMPS-NMR	SSO, links
CCP4 Online	Crystallography	ARP/wARP, PDB-REDO	SSO, links
PDB-REDO	Crystallography	3dBioNotes, CCP4Online, ARP/wARP	parallelised, new functionality, SSO, links
ARP/wARP	Crystallography/ EM	PDB-REDO, CCP4Online, DipCheck, ViCi, Auto-Rickshaw	redesign, EM, SSO, links
ProteinCCD	Crystallography		parallelised, new functionality, SSO
3DBionotes	Bioinformatics	HADDOCK, PDB-REDO	extended API, links
MetalPDB	Bioinformatics		database extended
ViCi	Crystallography/ Bioinformatics	ARP/wARP	links
AutoRickshaw	Crystallography	ARP/wARP	links

### 2.1 Portal

The principal entry point to access West-Life computational services is its web site at https://about.west-life.eu/. The site provides links to the component services, categorised by experimental or computational discipline. These services are run by individual West-Life partners, and are typically well established in their own right. Structural biologists can and do access these services directly, but access via West-Life provides additional integration tools, and helps new users to discover the services. Table 1 provides an overview of the component services. The West-Life website also provides links to various tutorials and webinars to learn how to make best use of the services.

The principal target audience for the portal is the international community of structural biologists, ranging from experienced scientists to young researchers. Those new to structural biology will benefit from the one-stop shop for computational services, while those already familiar with the available software can benefit from the new data management tools and the use of grid and cloud resources that improve performance over carrying out calculations in-house. The portal is also designed to be usable by other scientists who have an interest in understanding experimental structural data, or who wish to perform additional structural modelling or comparisons. Although the portal was developed with European funding, the West-Life portal and the services it links to are open to researchers worldwide.

### 2.2 Enhanced services

The project has enabled considerable improvements to the following web services for structure determination: AMPS-NMR (Bertini et al., 2011, Verlato et al., 2017), ARP/wARP (Langer et al., 2008), DisVis and PowerFit (van Zundert et al., 2017), FANTEN (Rinaldelli et al., 2014), HADDOCK (Vangone et al., 2017, Spiliotopoulos et al., 2016), MetalPDB (Putignano et al., 2018), PDB-REDO (Joosten et al., 2014), ProteinCCD (Mooij et al., 2009), and Scipion (Conesa Mingo et al., 2017, Cuenca-Alba et al., 2017). The changes made cover extensions to different experimental data and improvements in algorithms and usability.

West-Life has also participated in the development of new services: SpotOn for the identification of hot-spot residues in protein complexes (Moreira et al., 2017), PRODIGY for the prediction of binding affinities for protein-protein complexes (Xue et al., 2016), and protein-small ligand (PRODIGY-LIG) complexes (Vangone et al., 2018), 3DBionotes for annotating structures with biochemical and biomedical information (Segura et al., 2017), and Dipcheck for validating protein backbone geometry (Pereira and Lamzin, 2017), and new algorithms and a new service for ARP/wARP Classic EM to build models into EM maps. Development and improvements to community services supporting the prediction of macromolecular assemblies (Lensink et al., 2017), were carried out as part of the project, including provision of a server that provides validation of predicted macromolecular assemblies.

These services are summarised in Table 1, and details of the most significant enhancements follow.

• The **AMPS-NMR** (AMBER-based Portal Server for NMR structures) (Bertini et al., 2011) provides a user-friendly interface to perform energy refinement of experimental NMR structures by restrained molecular dynamics (rMD), as well as to run unrestrained molecular dynamics simulations. The service has been extended to use GPGPU cards to achieve faster calculations; in addition, the user authentication mechanism has been updated to adopt also the West-Life Single Sign On (SSO), which is discussed below.
• The **ARP/wARP** web service for MX protein model building and refinement has been available since 2004. By the end of 2017 about 40,000 model building jobs had been submitted remotely by almost 5,000 researchers. Within the West-Life project the web service was redesigned and a modern and newly employed web interface now provides remote access to all functionalities of the ARP/wARP software to the users worldwide. The service has been extended to accept cryo-EM maps as input for de novo model building and refinement and also has been updated to adopt the West-Life SSO.
• **DisVis** is a web service dedicated to the visualisation and quantification of the information content contained in distance restraints between macromolecular complexes. Given a set of distance restraints coming from experiments (crosslinks, FRET, etc.), DisVis systematically tests all combination of distances to report incoherent distance, potential false-positives or multiple interaction modes. DisVis relies on a parallel implementation of Fast Fourier Transform that can profit from multi-CPU or GPU resources (van Zundert et al., 2017).
• **FANTEN** is a web tool for the determination of the anisotropy tensors related to NMR-measured pseudocontact shifts and residual dipolar couplings. Within West-Life, FANTEN has been connected to the Virtual Folder for cloud storage. A direct connection to the HADDOCK server has been implemented, allowing FANTEN-generated models of protein-protein adduct to be directly submitted to HADDOCK for flexible refinement.
• Given a cryo-EM map and an atomic model, **Powerfit** can fit the model to the map with relatively high accuracy (van Zundert, 2015) while it is very fast in terms of computational speed. A webserver has been also developed and made available under the West-Life portfolio (van Zundert et al., 2017).
• **HADDOCK** is a well-established and popular program for integrative modelling of various types of biomolecular complexes. It can make use of a wide variety of experimental data as restraints to guide the docking. Under West-Life, it has been extended to include cryo-EM density maps as an additional restraints. The Powerfit fitting algorithm is included as the first step to establish likely component locations, before full docking and refinement (van Zundert et al., 2015).
• **MetalPDB** (http://metalweb.cerm.unifi.it/) is a database providing information on metal-binding sites detected in the three-dimensional (3D) structures of biological macromolecules. MetalPDB represents such sites as 3D templates, called Minimal Functional Sites (MFSs), which describe the local environment around the metal(s) independently of the larger context of the macromolecular structure. MetalPDB includes a variety of tools for the structural comparison of sites, both pairwise or against the full database. Within West-Life, the database was extended significantly, introducing a number of new features (Putignano et al., 2018).
• **PDB-REDO** provides an automated procedure to optimise structure models from MX. Many decision-making algorithms in the PDB-REDO pipeline use comparative, statistical analysis of alternative models of the crystallographic data. These algorithms contribute strongly to its performance in terms of model quality, but they also make the procedure time consuming. The PDB-REDO procedure has been parallelized, giving an average 4-fold speedup with no compromise to the quality of the model, and easing the incorporation of PDB-REDO in other services.
  After analyzing the entire PDB for homologous models and mining relevant data, the PDB-REDO databank of *redone* Protein Data Bank (Berman, 2000) entries, was completely replaced and integrated into the PDB-REDO webserver thus allowing it to serve as a data source to other West-Life services. The new homology-based functionality in PDB-REDO was incorporated (van Beusekom et al., 2018). Implementing additional algorithmic developments of PDB-REDO components and catalyzing enhancements in third-party software such as REFMAC (Murshudov et al., 2011) has lead to an overall 10-fold speedup of the PDB-REDO procedure. The West-Life Single Sign On was added as an authentication method.
• **PRODIGY** and **PRODIGY-LIG** are web servers that predict binding affinities for protein-protein (PRODIGY) and protein-small ligand (PRODIGY-LIG) complexes. To use PRODIGY one just needs to provide the three-dimensional structure of complex/complexes in PDB/mmCIF format or the ID of its PDB entry. In the associated study (Vangone and Bonvin, 2015), it has been shown that the number of interfacial contacts (ICs) of a protein-protein complex correlates with the experimental binding affinity. This information, combined with properties of the non-interacting surfaces (NIS), which have been shown to influence binding affinity, has led to one of the best performing predictor so far which was also benchmarked on such a large and heterogeneous set of data. Depending on the input data to be used, a request is expected to take a few seconds before a user receives the results.
• **ProteinCCD** facilitates the informed design of several truncation constructs of a protein under investigation, enabling researchers to achieve successful expression and crystallisation for MX. The service has been implemented in a new computational platform allowing several improvements, including: parallel processing of server requests, more efficient interface for construct design, more cloning methods, an extended collection of existing vectors, local execution of some algorithms for improving response time, new servers for meta-analysis, direct data base retrieval of isoforms of a gene under investigation, easy bookkeeping, and better data security. New algorithms enable the ranking of constructs, reflecting their chances to yield high quality soluble protein likely to result in a successful structural determination experiment. A quality score is assigned which is returned to the user.
• **Scipion** is an image processing framework to obtain 3D models of macromolecular complexes using electron microscopy. Normally, Scipion is run locally, but some functionality is made available via Scipion Web Tools and these services have been substantially enhanced under West-Life. Furthermore these services have been modified to accept input data from the WestLife VirtualFolder. In addition, work has been done towards automatic deployment in the EGI Federated Cloud of the full Scipion application through installation with Cloudify recipes or using predefined appliances.
• **3DBIONOTES** (Segura et al., 2017) a web framework developed to integrate structural data with genomics, proteomics and interactomics information. The application displays biomedical and biochemical annotations that can be mapped on proteins including their sequence and structure, gene sequences and protein-protein interaction networks. The API has been extended in order to provide new services for the programmatic submission of structural data and annotations. In this way, other web resources as HADDOCK or PDB-REDO can display their data and results through a web browser.
• **CAPRI** is a structural biology community-wide experiment, developed to assess the quality of predicted macromolecular assemblies (Wodak and Janin, 2017). Prediction of macromolecular assemblies is an active field of research, with CAPRI being at the forefront of bringing the community together and in coordinating the activities to help development of computational approaches. A search system for predicted macromolecular assemblies has been developed, as well as a web-service allowing predictors to assess the quality of their macromolecular models to support rapid advances in computational approaches for modeling large macromolecular machines (See Quality Assurance below).

### 2.3 Quality Assurance

There are many processing steps from experimental data to the structure. Some solely use the experimental data, some use prior knowledge about macromolecular structures, and some involve the scientist’s judgment. Validation of the final structure is important, to ensure it is consistent with all experimental data and with known features of macromolecules. West-Life has contributed to the development of specific validation tools. For example, it enabled work on validation of rigid-body fitting of cryo-EM maps (van Zundert and Bonvin, 2016) and participated in evaluation activities (Kurkcuoglu et al., 2018). Scipion Web Tools has implemented methods for assessing the reliability of a 3D cryo-EM reconstruction against the original set of 2D particle images, and new methods for estimating local resolution have been added to an existing service (Conesa Mingo et al., 2017). The new web service DipCheck (http://arpwarp.embl-hamburg.de/) provides validation criteria for protein backbone geometry which is independent of the conventional Ramachandran plot. Additionally, the validation method developed during CAPRI evaluation meetings over almost two decades (Wodak and Janin, 2017) has been implemented as a web-service (http://www.ebi.ac.uk/pdbe/complex-pred/capri-validation/) to allow users to assess the quality of their predicted macromolecular complexes, based on established standards involving both geometric and biological properties of the models.

In addition to these specific tools, the pipelining and metadata activities of West-Life (see below) enable and encourage the cross-validation of structures against other tools and metrics. For example, the PDB-REDO service provides a standard refinement of crystallographic structures, and is now easily accessible through pipelines from third party web services. Wider validation of structural results can also be provided by closer linking to the biological context, as is provided by the West-Life service 3DBionotes. In addition, a West-Life pilot project looked at the use of text mining for retrieving structural information from associated publications. The West-Life consortium worked with EuropePMC to identify mentions of specific protein residues, and cross-reference these to the Uniprot entries associated with the structure.

### 2.4 Pipelines

**Fig. 3:**
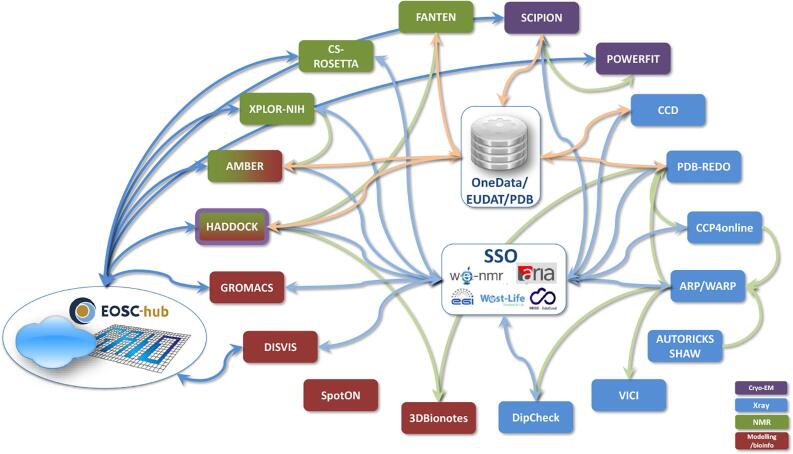
A schematic representation of the pipelines implemented by West-Life. Grey lines indicate direct interactions between services, green lines indicate interactions with centralised data stores, and purple lines indicate interactions with SSO services. (For interpretation of the references to colour in this figure legend, the reader is referred to the web version of this article.)

The project has facilitated the sequential use of multiple web services by building direct connections between portals, see Fig. 3 and Table 1. For example, CCP4 Online provides Balbes (Long et al., 2008), MrBUMP (Keegan et al., 2018) and MoRDa (Vagin and Lebedev, 2015) services for solving crystal structures via molecular replacement. The output coordinate model should be correctly placed, but as a first model may not be sufficiently accurate for subsequent standard refinement to proceed. West-Life pipelines enable this partial solution to be sent to the ARP/wARP (Langer et al., 2008) or PDB-REDO (Joosten et al., 2014) services for further building and refinement.

Construction of such pipelines varies with the specific services involved, but has typically required the development or improvement of programmatic access to allow the seamless transfer of data and metadata from one service to another. Since many services are computationally intensive and may require hours to complete, the ability to transfer data autonomously is very important. The adoption of Single Sign On mechanisms (see below) was also crucial, avoiding the requirement for the researcher to login separately to individual web services.

### 2.5 Data management

Typically, a structural biology project accumulates a large number of data files associated with different instruments and different pieces of software. Managing these data throughout the project can be difficult, and returning to a project or inheriting someone else’s project at a later date even more so. West-Life has attempted to alleviate these potential problems through the development of two new facilities for data management.

The West-Life Virtual Folder addresses the issue of accessing data scattered among different data storage providers. It is a web-based application allowing a user to register data storage providers and connect accounts from multiple places in one common interface. The data are not physically aggregated, since many datasets can be large. Instead, the application allows a user to view all data and download selected data on request, or to obtain a publicly accessible link conforming to the WEBDAV protocol to either a particular file or a particular folder which can then be used by another tool to download, process and upload results. Currently Dropbox and WebDAV based data storage providers are supported (e.g. EUDAT B2DROP service, ownCloud instances with WebDAV enabled or Pcloud commercial service).

Aside from allowing access to sequence or structure data, the Virtual Folder also allows visualization of structures in a web-based 3D viewer LiteMol (Sehnal et al., 2017). Additional web-based visualisations for sequence data and value added annotation, alongside an interactive display of topology for a particular protein molecule, allows users better understanding of protein structures thus making it easy for a non-specialist user to understand the 3D structures available in the PDB. The web based components use the PDBe REST API (pdbe.org/api) to access information from PDBe database.

INFN has made available a Onedata experimental storage space to all West-Life users. Onedata (http://onedata.org) is a global data access solution for science developed by the AGH University of Science and Technology in Poland as part of the INDIGO-DataCloud project (Salomoni et al., 2018), and currently supported by EOSC-Hub and XDC European projects. With Onedata, users can perform heavy computations on huge datasets, access their data in a dropbox-like fashion regardless of its location, publish and share their results with public or closed communities. Even if at the time of writing only a release candidate of Onedata software is available, West-Life developers managed to integrate West-Life SSO as authentication method. Moreover, a plugin prototype for using Onedata as storage back-end for the Virtual Folder has also been developed. In the context of EOSC-Hub and XDC project, INFN has committed to keep updated with the evolution of Onedata software and continue in providing up to 10 terabytes of storage in Onedata.

The West-Life Repository addresses the issue of the lack of support for data management during data acquisition at smaller facilities. Large facilities often have a full solution for data management supporting the full lifecycle of the projects, performing experiments and enabling access to data during and after a visit via a uniform interface. The West-Life Repository is a lightweight exemplary implementation of these procedures, suitable for deployment at small facilities. The current implementation integrates the West-Life SSO, so that a user visiting a facility does not need to create a special account. It also integrates the ARIA system to allow users to import existing project proposal data from the Instruct website (https://www.structuralbiology.eu).

### 2.6 Metadata

Structural biology has a long tradition of open data, notably through depositing structures in the Protein Data Bank, which opened as early as 1971 (Crystallography: Protein data bank, (1971), Bernstein et al., 1977, Berman, 2000).

Nevertheless there are challenges to full compliance with FAIR principles (Wilkinson et al., 2016). Current workflows in Structural Biology may not always be properly described in an unambiguous manner due to a combination of lack of metadata reporting and lack of appropriate metadata standards. There is no agreed ontology for the primary data processing, either at the level of integrated studies combining different technologies or even at the single technique level. Metadata about experimental conditions are often incomplete. As a result of this, the chain of custody from sample to publication is often broken at several points. The greatest challenges however lie not in devising new metadata standards, but in securing the use of existing ones.

Most West-Life partners extended their existing services from the PDB file standard to accept and export PDBx/mmCIF files, the master format for PDB archive representing macromolecular structure data, which enables the processing of larger structures and the incorporation of more metadata. PDBx/mmCIF is emerging as the standard in structural biology, as most refinement packages and data archived have adopted mmCIF as their data definition and exchange format. Additionally, most West-Life partners evaluate or process their input based on well-established dictionaries, such as the PDB Exchange Dictionary, NMR Exchange Format Dictionary, Small Angle Scattering Dictionary, and others. Scipion evaluated the CWL standard for describing workflows (https://www.commonwl.org/v1.0/). As an additional work, Scipion has implemented direct EMPIAR submission to try to encourage sharing acquisition data from facilities.

**Fig. 4:**
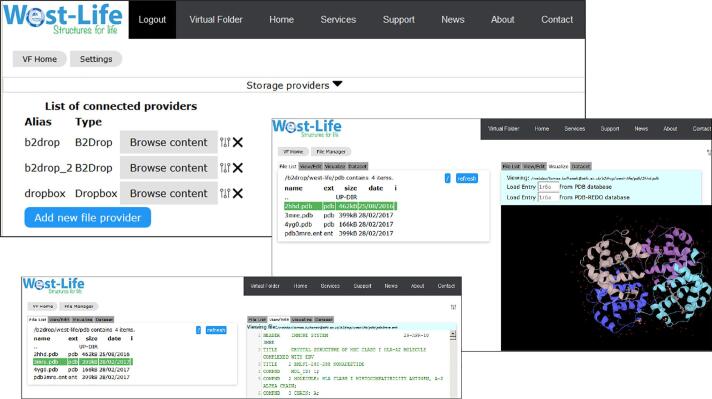
An instance of the Virtual Folder, accessible from the West-Life VRE portal after login. Three connected data providers are shown, together with a file listing from one. Standard file browsing utilities are available, together with bespoke tools such as a PDB viewer.

The Virtual Folder, described above and illustrated in Fig. 4, supports the PROV-O ontology for tracing the provenance of a paper, back through the processing steps, to the structural experiment (https://www.w3.org/TR/2013/NOTE-prov-overview-20130430/).

### 2.7 Single Sign On

The advances described could be less attractive if use of each service required service-specific login credentials. The West-Life project considered it essential to implement a system for Single Sign On (SSO) and identity management. The design of West-Life SSO follows the latest developments in the area, and it is compatible with the current state of the art. The system provides authentication and authorization functions, which together with an identity management system forms the security layer of West-Life.

The core of the West-Life SSO system is an “IdP-SP proxy” that links identity providers such as universities and social networks with the registered services. West-Life users are not required to maintain a new set of credentials, instead they continue to use their existing authentication services, with the proxy playing the unifying role in the process. The services register only with the West-Life proxy regardless how many authentication providers are supported. The proxy uses standard protocols like SAML and OIDC for communication with end services.

The proxy service is linked with the identity management system that maintains the users of West-Life services. The system supports the whole life cycle of users, from the registration procedure to potential cease of the membership. When users register, they assent to an appropriate Acceptable Use Policy, and West-Life partners worked together to conform to the new European General Data Protection Regulations. The system maintains set of attributes for every user, which is made available to the end service. User registered with the management system can be arranged in groups to ease maintenance and provide the basis for access control.

There are several initiatives in Europe today to implement SSO for researchers, so one design goal was to be ready to integrate with them. The result is compatible with on-going efforts in the life-science domains, in particular the initiative of ELIXIR, and is ready to utilize the outcomes of the activities, which will improve sustainability of the whole solution.

### 2.8 EGI

Many of the West-Life services run on the European Grid Infrastructure (EGI), submitting several million jobs a year consuming around 20 million CPU hours, as normalized by HS06, a HEP-wide benchmark for measuring CPU performance based on SPEC® CPU2006 benchmark suite. The EGI extended a Service Level Agreement with the predecessor project WeNMR to December 2020, committing up to 53 million CPU hours and 54 terabytes of storage by five resource centers in the Netherlands, Italy, Portugal and Taiwan. However, during the lifetime of the project the computing workload has been distributed across more than thirty EGI sites in Europe, America and Asia. The provision includes GPU resources (Verlato et al., 2017), which can be used for example by the West-Life AMPS-NMR, DisViS and Powerfit services. EGI also committed Federated Cloud resources supported by two sites in Czech Republic and Italy, with up to 160 virtual CPUs and up to 4 terabytes of storage. From its beginning, West-Life applications have consumed over 57 million CPU hours provided by the EGI HTC infrastructure, and executed over 4000 Virtual Machines for more than 700,000 h on the EGI Federated Cloud.

### 2.9 Virtualized portals

Despite the advantages of centralised web portals provided as a service to the community, this approach can be limited by the lack of user control, the impossibility to fine-tune the application, and problems with resolving contradicting requirement of different users. This depends on the options exposed to users on a specific portal. Some might offer a very simple interface with few options for fine-tuning, while others, for example the Guru interface of the HADDOCK portal, allow experienced users to control over 500 different parameters. Most users in general only access a limited number of options, but for challenging projects or particular experiment types more control can be useful.

Some of these problems can be solved with “portal virtualisation”. Instead of setting up a single instance of the application portal, a detailed “recipe” to deploy the portal in a cloud environment is provided. Then a group of users with specific needs can take this recipe, make minor modifications to it, and execute it using available cloud orchestration services. The result is a customised portal being set up with minimal effort, and the whole process is easily repeatable.

We developed a framework to adapt virtually any service to this setup (http://internal-wiki.west-life.eu/index.php/Cloud_Orchestration_Training).

As a concrete example, the Scipion portal developed by West-Life uses this framework. Although Scipion Web Tools provides a set of services to try some of the workflows most used in cryoEM, users might want to try the whole functionality of Scipion. This is now possible by deploying this portal in the EGI Federated Cloud using the framework (https://github.com/ICS-MU/westlife-cloudify-scipion). Besides installing the Scipion software and all its dependencies, the deployment can make use of a Nvidia GPU, when available in the cloud machine, for both computation and accelerated 3D rendering through VirtualGL.

## 3 Conclusions

Structural biology is a very diverse field, making top-down solutions impractical to implement. This is particularly true of software, where innovative algorithms and data analysis approaches can gain popularity quickly. The approach of West-Life has therefore been to address interoperability between computational services rather than, for example, imposing a single data model or workflow engine. Thus the work described above covers enhanced services, pipelining across services and quality assurance.

In addition to the provision of tools, a structural biology environment requires substantial compute resources and ways to handle large and diverse datasets. Thus, West-Life has also worked on the provision of Grid and Cloud resources, in a way largely invisible to the user, and on data management tools.

*Outlook* West-Life has begun the task of building the information technology infrastructure required by hybrid approaches to structural biology that use combined techniques. It has established a platform of interoperable services, open for new services, and without a central point of management. Instruct has committed to sustaining appropriate components of this infrastructure. Some portals are part of the Thematic Services provided by the EOSC-hub initiative (https://marketplace.eosc-hub.eu/46-wenmr-suite-for-structural-biology). Other services will be sustained and extended by the institutions that developed them.

## 4 Materials and methods

All new codes produced by the project are open source, and are available on Github athttps://github.com/h2020-westlife-eu/. Where developments are extensions to existing codes, they are conformant to the existing licenses, which give free use for academic purposes.

## Conflict of interest

None.
